# Digital Phenotyping of Anxiety–Depression Comorbidity in Tele–Mental Health: Severity Coupling and Resource-Use Signatures in a Real-World Cohort

**DOI:** 10.3390/medsci14030368

**Published:** 2026-07-02

**Authors:** Anastácia Zoriy, Ana Dionísio, Filipe Pinto, Nuno Vale

**Affiliations:** 1 PerMed Research Group, RISE-Health, Faculty of Medicine, University of Porto, Alameda Professor Hernâni Monteiro, 4200-319 Porto, Portugal; up202006025@edu.med.up.pt; 2 RISE-Health, Department of Community Medicine, Health Information and Decision (MEDCIDS), Faculty of Medicine, University of Porto, Rua Doutor Plácido da Costa, 4200-450 Porto, Portugal; filipe@knokcare.com; 3 Knok, Rua Mouzinho de Albuquerque 742, 4450-007 Matosinhos, Portugal; ana.dionisio@knokcare.com; 4 Laboratory of Personalized Medicine, Department of Community Medicine, Health Information and Decision (MEDCIDS), Faculty of Medicine, University of Porto, Rua Doutor Plácido da Costa, 4200-450 Porto, Portugal

**Keywords:** telepsychiatry, telemedicine, digital phenotyping, anxiety, depression, comorbidity, severity stratification, real-world data

## Abstract

**Background**: Anxiety and depression are major contributors to mental-health burden and frequently co-occur in clinical practice. In tele–mental health, routinely captured operational variables such as consultation duration, visit frequency, and follow-up cadence may provide clinical digital phenotypes that complement conventional symptom scales. This study aimed to characterize anxiety–depression comorbidity in a large real-world tele–mental health cohort and to determine whether symptom severity was associated with distinct patterns of healthcare utilization. **Methods**: We conducted a retrospective real-world study of 3467 patients followed in psychiatry and psychology teleconsultations. Patients were classified as anxiety only, depression only, comorbid anxiety–depression, or neither. Symptom severity was categorized as mild, moderate, or severe using validated questionnaire-based measures; to improve comparability across instruments, scores were additionally harmonized using z-score normalization. Associations between anxiety and depression severity within the comorbid subgroup were examined using a chi-square framework. Telehealth utilization endpoints included consultation duration, number of consultations, and inter-visit interval, analysed overall and stratified by sex, age group, and symptom severity. **Results**: Anxiety and/or depression were present in 61.7% of the cohort (2140/3467), and anxiety–depression comorbidity accounted for 43.8% of all patients (1520/3467), indicating substantial real-world overlap. Within comorbid cases, anxiety and depression severity were strongly coupled, with depression severity varying systematically across anxiety severity strata (chi-square *p* = 9.88 × 10^−102^). Compared with isolated anxiety or depression, comorbidity was associated with a more intensive healthcare-utilization profile, characterized by a higher mean number of consultations and shorter inter-visit intervals. Among comorbid patients, females showed greater longitudinal service use than males, with more visits and closer follow-up. Resource use also varied according to symptom burden, mainly in depression, supporting a graded relationship between clinical severity and operational care demand. **Conclusions**: In this large real-world tele–mental health cohort, anxiety–depression comorbidity was highly prevalent, clinically structured, and associated with distinct and measurable resource-use signatures. These findings highlight the novelty and practical value of integrating symptom severity with operational telehealth data to derive pragmatic digital phenotypes of care intensity. Such phenotypes may support risk stratification, triage, follow-up scheduling, and capacity planning in tele–mental health, with potential translational relevance for broader mental healthcare systems. However, these findings should be considered descriptive and hypothesis-generating and warrant further longitudinal validation in other clinical settings.

## 1. Introduction

Anxiety and depressive disorders are among the most prevalent and disabling mental-health conditions worldwide. According to the World Health Organization, approximately 4.4% of the global population was living with depression and 3.6% with anxiety disorders in 2015 [1]. The Global Burden of Disease Study 2019 further demonstrated that mental disorders account for a substantial proportion of years lived with disability (YLDs), with depressive disorders ranking among the leading causes of disability across age groups [2]. Although age-standardized rates have remained relatively stable over time, the absolute number of affected individuals continues to increase due to demographic expansion and population ageing [2,3]. Projections suggest that the global burden of depression will continue to rise by 2030, reinforcing the need for scalable and efficient models of care delivery [3]. Beyond their clinical burden, depressive disorders are also associated with substantial economic costs related to healthcare expenditure, productivity losses, and disability worldwide [4]. In Portugal, epidemiological data indicate particularly high prevalence rates of anxiety disorders (16.5%), followed by depressive disorders (7.9%) [5]. These figures illustrate the magnitude of anxiety- and affective-related conditions in the national context and reinforce their importance as a priority area for public health intervention. Mental and behavioural disorders account for a significant proportion of healthcare utilization and public expenditure, with depression being a major driver of disability, absenteeism, and early retirement [6]. Moreover, both anxiety and depressive disorders are frequently associated with chronicity, recurrence, and functional impairment, thereby increasing the need for sustained clinical follow-up and efficient care coordination. Their impact is therefore not limited to individual suffering, but also extends to families, employers, and the wider healthcare and social support systems. In this context, optimizing care pathways for anxiety and depression is of clear clinical and socioeconomic relevance, particularly when supported by care models capable of improving access, continuity, monitoring, and personalization of care.

Although traditionally conceptualized as distinct diagnostic entities, anxiety and depression frequently co-occur, sharing overlapping symptom profiles and neurobiological mechanisms [7,8]. Comorbidity between these disorders is common and clinically meaningful, being associated with greater symptom severity, poorer treatment response, higher chronicity, and increased suicide risk compared with single diagnoses [9,10,11]. Contemporary studies have consistently shown that a substantial proportion of individuals with major depressive disorder also meet criteria for at least one anxiety disorder during their lifetime [12]. Contemporary transdiagnostic models emphasize shared affective and cognitive vulnerabilities, supporting a dimensional rather than purely categorical understanding of internalizing psychopathology [12,13]. Earlier theoretical frameworks similarly proposed that anxiety and depressive disorders share common etiological mechanisms while maintaining partially distinct symptom dimensions [13]. The DSM-5 reflects this overlap through the “anxious distress” specifier in major depressive disorder, highlighting the prognostic relevance of anxiety symptoms within depressive episodes. Individuals with anxious depression often present greater functional impairment and lower responsiveness to treatment [11]. Thus, examining anxiety–depression comorbidity through a severity-based framework may provide more clinically informative insights than categorical diagnosis alone.

Sex and age differences may also influence patterns of symptom severity and healthcare utilization. Women consistently show higher prevalence and comorbidity rates for anxiety and depression [14,15,16]. Meta-analytic evidence indicates that women are approximately twice as likely as men to develop depressive disorders [16]. Several biological and psychological mechanisms have been proposed to explain these differences, including hormonal influences, stress reactivity and differential exposure to psychological stressors [17]. Age-related variations in symptom presentation and chronicity may further affect follow-up needs. Therefore, demographic factors should be considered when examining the association between symptom severity and operational healthcare metrics.

Over the past decade, tele–mental health has become an increasingly adopted modality for delivering psychiatric and psychological care. Telemedicine interventions have demonstrated clinically meaningful reductions in depressive and anxiety symptoms across multiple populations [18,19,20]. Systematic reviews and meta-analyses have reported that telepsychiatry can achieve outcomes comparable to face-to-face care while improving accessibility and continuity of treatment, particularly for populations facing geographic or logistical barriers to mental health services [18,19,20,21]. More recent evidence further supports the effectiveness of telemedicine interventions for managing depression and anxiety across different age groups, including older adults [22].

Beyond therapeutic effectiveness, telehealth systems generate structured and longitudinal datasets that integrate symptom severity assessments with operational metrics, such as consultation duration, number of visits, and inter-visit intervals. Unlike traditional care settings, where operational data are often fragmented, inconsistently recorded, or difficult to aggregate across encounters, tele–mental health platforms routinely capture standardized clinical and service-level information in a format that is more readily amenable to analysis. This creates a valuable real-world data environment in which symptom trajectories can be examined alongside patterns of care delivery and healthcare utilization. As a result, these systems make it possible to explore not only whether patients improve over time, but also how different levels of symptom burden may be associated with consultation frequency, monitoring intensity, and follow-up dynamics. Such an integrated perspective is particularly relevant in mental health, where chronicity, recurrence, and fluctuating severity often require repeated contacts and individualized care pathways. In this sense, telehealth platforms offer a unique opportunity to connect clinical status with operational demand, thereby supporting a more nuanced understanding of how symptom burden may translate into service utilization in routine practice.

Digital phenotyping has recently emerged as a framework for characterizing clinically meaningful behavioural and clinical patterns using routinely collected digital data [23,24,25]. In tele–mental health environments, validated symptom scales—such as the Patient Health Questionnaire-9 (PHQ-9) for depression [26] and the Beck Anxiety Inventory (BAI) for anxiety [27]—are systematically recorded alongside operational service metrics. This combination allows the examination of whether symptom severity translates into distinct patterns of healthcare utilization.

From a clinical-services perspective, consultation duration, visit frequency, and follow-up intervals may reflect underlying symptom burden, treatment complexity, or monitoring needs. In this context, anxiety–depression comorbidity may manifest not only as increased symptom severity but also as identifiable “resource-use signatures.” These operational patterns may function as clinically interpretable digital phenotypes embedded within real-world care delivery systems, offering potential value for stratified follow-up planning and resource allocation. In this context, the present study does not aim to validate digital phenotypes per se, but rather to explore whether symptom severity and comorbidity are associated with reproducible healthcare-utilization profiles within a real-world tele–mental health environment.

Despite extensive literature documenting the high prevalence and clinical impact of anxiety–depression comorbidity [7,8,9,10,11,12], relatively few studies have integrated dimensional symptom severity with longitudinal healthcare utilization metrics in real-world tele–mental health cohorts. Most existing research relies on cross-sectional epidemiological data or controlled clinical trials, with limited attention to how severity coupling translates into patterns of service use within routine clinical practice.

The present study aims to address this gap by examining anxiety–depression comorbidity within a large real-world tele–mental health cohort. Specifically, we investigate whether increasing symptom severity and comorbidity are associated with differences in consultation duration, number of visits, and inter-visit intervals. In addition, we explore whether these patterns vary according to age group and sex in order to identify demographic-stratified digital phenotypes of healthcare utilization. By linking validated symptom measures to operational healthcare metrics, this study seeks to characterize clinically meaningful resource-use signatures that may support risk stratification and optimize follow-up planning in tele–mental health settings, with potential applicability beyond digital care environments. The innovative value of this study lies in its integration of two domains that are commonly examined in isolation: psychiatric symptom burden and the operational architecture of care delivery. Rather than focusing exclusively on symptom change, we examine how anxiety, depression, and their co-occurrence may be reflected in measurable patterns of service use in routine clinical practice. This approach is particularly relevant in tele–mental health, where longitudinal, standardized, and platform-native data allow the simultaneous evaluation of clinical severity and healthcare utilization at scale. By capturing not only whether patients present with anxiety or depression, but also how these conditions translate into consultation intensity, monitoring frequency, and follow-up spacing, the study advances a more granular understanding of care demand in real-world mental health services.

Importantly, the study also addresses a clinically meaningful and often under-characterized dimension of psychiatric care by focusing on anxiety–depression comorbidity rather than on isolated diagnostic categories alone. Because these disorders frequently overlap, and because such overlap may be associated with greater complexity, persistence, and service needs, a comorbidity-informed framework may offer greater clinical realism than single-disorder models. The additional stratification by age and sex further strengthens the analysis by allowing the identification of subgroup-specific utilization patterns, thereby supporting a more personalized and data-informed perspective on care delivery. The present study should be viewed primarily as a descriptive analysis of symptom burden and healthcare utilization within a large tele–mental health cohort. Building on the growing interest in the use of routinely collected healthcare data to characterize patterns of service use, the study explores whether clinically relevant associations emerge from the joint evaluation of symptom severity, comorbidity status, and operational care metrics. While such patterns may ultimately contribute to a better understanding of care demand and support future efforts in resource planning and care pathway optimization, the present analysis does not aim to establish predictive phenotypes, validate digital phenotyping approaches, or evaluate specific service-planning interventions. Instead, it seeks to identify and describe healthcare-utilization profiles that may inform future longitudinal and predictive research in tele–mental health settings. Although the findings should be interpreted as exploratory and hypothesis-generating, the identification of reproducible utilization patterns may represent an important preliminary step toward the future development of operational or clinically informed digital phenotypes. Further research integrating longitudinal outcomes, treatment trajectories, and additional behavioural or healthcare-use data will be necessary to determine whether these profiles can evolve into robust digital phenotypes capable of supporting personalized care and service planning. In this respect, the study has translational relevance for both telemedicine-based models and conventional mental healthcare systems seeking to incorporate data-driven approaches into patient stratification and care pathway optimization.

## 2. Materials and Methods

This retrospective observational cohort study was based on routinely collected real-world clinical data from a tele–mental health platform. The study aimed to examine the association between anxiety–depression severity, comorbidity patterns, and healthcare utilization metrics within a naturalistic care setting.

Data were provided by knok Healthcare (Matosinhos, Portugal), a Portugal-based telemedicine provider delivering consultations across multiple specialties, including Psychiatry and Psychology. The dataset comprised anonymized clinical records from patients who attended at least one Psychiatry or Psychology teleconsultation between 16 February 2021 and 7 April 2025. Patient-reported questionnaire data were available from 19 June 2023 to 7 April 2025. Consequently, symptom-severity measures may not fully capture patients’ clinical status throughout the entire healthcare-utilization observation period. All records were fully anonymized prior to analysis. All patients with at least one registered Psychiatry or Psychology consultation during the study period were eligible for inclusion. The final sample included 3467 unique patients, corresponding to 25,696 consultations and 4661 completed symptom questionnaires. Patients of all age groups and both sexes were included, and no additional exclusion criteria were applied. Prior to analysis, data were reviewed for internal consistency, including verification of consultation dates, consultation duration, inter-visit intervals, and demographic variables. No implausible values requiring exclusion were identified, and all available observations were retained for analysis.

Sociodemographic variables included biological sex (male/female) and age, which was treated as a continuous variable in the analyses. The primary operational outcomes were mean consultation duration (in minutes), total number of consultations per patient and inter-visit interval (defined as the median number of days between consecutive consultations). To address potential confounding arising from differences in follow-up duration, an additional utilization metric was calculated. Consultation rate was defined as the average number of days per consultation during the observed follow-up period. Lower values indicate more frequent clinical contacts relative to follow-up duration. This measure was analysed across sex, age groups, diagnostic categories, and symptom-severity strata as a complementary follow-up-adjusted indicator of healthcare utilization.

Consultation duration was calculated using automatically recorded start and end timestamps and inter-visit intervals were computed based on consecutive appointment dates. Clinical diagnoses were coded by the consulting healthcare professionals according to the International Classification of Diseases (ICD). The categories ‘anxiety only’, ‘depression only’, ‘anxiety–depression comorbidity’, and ‘none’ were defined exclusively using clinician-recorded ICD diagnoses extracted from the electronic health record, whereas symptom severity was assessed separately using validated questionnaire measures. Anxiety severity was evaluated using Beck Anxiety Inventory (BAI), DASS-21 Anxiety subscale or Generalized Anxiety Disorder-7 (GAD-7). Depressive symptoms were assessed using Patient Health Questionnaire-9 (PHQ-9), Beck Depression Inventory (BDI) or DASS-21 Depression subscale. Different questionnaires were administered across individuals, reflecting routine clinical practice, with each patient completing one or more of the available measures for each domain. Severity analyses were restricted to patients with available questionnaire data. Consequently, although the full cohort comprised 3467 patients, severity-based analyses included approximately 1460–1470 individuals depending on the symptom domain analysed. This difference in sample composition should be considered when interpreting severity-related findings and may introduce selection bias. Additional psychiatric domains included stress, insomnia, suicidal risk, eating disorders, and burnout, defined using clinician-recorded diagnostic information extracted from the electronic health record and were not based on questionnaire-derived severity thresholds. Quality-of-life measures were excluded from comorbidity definitions. Raw questionnaire scores were categorized into severity levels (mild, moderate, severe) according to established cut-off values for each instrument. To ensure comparability across instruments measuring the same construct, standardized z-scores were calculated for anxiety and depression severity. Nevertheless, the use of multiple validated instruments with different psychometric characteristics may have introduced some degree of measurement heterogeneity, and severity-related findings should therefore be interpreted with appropriate caution. For patients with multiple instruments assessing the same domain, severity categorization followed the highest available validated score during the study period. When multiple instruments assessing the same symptom domain were available for a given patient, severity classification was based on the highest validated score recorded during the study period. This approach was chosen to maximize sensitivity to clinically relevant symptom burden; however, it may reflect peak rather than baseline severity and should therefore not be interpreted as a temporal predictor of subsequent healthcare utilization. In addition, because symptom questionnaires are relatively time-consuming to administer in routine clinical practice, most patients completed only one or two assessments during the study period. As a result, longitudinal symptom data were limited, restricting our ability to perform a finely stratified temporal analysis of symptom severity trajectories or to evaluate changes in severity over time in relation to healthcare utilization patterns. Sensitivity analyses stratified by individual questionnaire type were not performed. Within the present cohort, a single instrument predominated for each symptom domain (BAI for anxiety and PHQ-9 for depression), resulting in highly unbalanced instrument-specific subgroups. Consequently, such analyses were considered unlikely to provide meaningful additional insights and would have substantially reduced the interpretability of comparisons across instruments.

Continuous variables were summarized using means and standard deviations (SD), whereas categorical variables were described using absolute and relative frequencies. Within the comorbid subgroup, the association between anxiety and depression severity categories was assessed using the chi-square test of independence. To complement significance testing, effect size was estimated using Cramér’s V, and adjusted standardized residuals were calculated for individual cells in order to identify the combinations contributing most strongly to the overall association; residuals with absolute values of 2 or greater were considered to indicate meaningful deviations from expected frequencies.

Differences in operational outcomes, including consultation duration, number of consultations, and inter-visit interval, were evaluated across diagnostic groups and symptom-severity strata using analysis of variance (ANOVA). Given the large sample size, parametric inference was considered appropriate for the main comparisons, with particular emphasis on the overall robustness of ANOVA under moderate departures from normality. When global tests indicated statistically significant differences, post hoc pairwise comparisons were performed as appropriate, with adjustment for multiple testing to reduce the risk of type I error. Statistical significance was defined as a two-sided α level of 0.05. This analytical strategy was designed to examine both the categorical relationship between anxiety and depression severity within the comorbid subgroup and the extent to which clinical burden was associated with measurable differences in healthcare-utilization patterns. By combining association testing, effect-size estimation, and group-based comparisons of operational metrics, the statistical analysis enabled a structured evaluation of the relationship between symptom burden and service use in this real-world tele–mental health cohort.

All data were fully anonymized prior to analysis and contained no personally identifiable information, thereby ensuring that no individual participant could be directly or indirectly identified from the study dataset. Data handling procedures were conducted in accordance with applicable data protection and confidentiality requirements, including the General Data Protection Regulation (GDPR) and the institutional standards governing the secondary use of routine-care health data. The study was based exclusively on the retrospective secondary analysis of anonymized data generated during routine clinical care, with no direct contact with patients and no impact on clinical decision-making or ongoing treatment. Because the dataset had been irreversibly anonymized prior to access and analysis, and because the study did not involve any intervention, re-identification procedure, or collection of additional patient-level information, individual informed consent was not required under the applicable ethical and regulatory framework.

This approach ensured that the study could generate clinically relevant real-world evidence while preserving patient privacy, data security, and compliance with current legal and ethical standards for the use of health-related data in observational research.

## 3. Results

### 3.1. Sample Characteristics

The final cohort comprised 3467 patients, corresponding to 25,696 tele–mental health consultations and 4661 completed symptom questionnaires, thereby providing a substantial real-world dataset for the evaluation of both clinical burden and operational care patterns. Participants were predominantly young adults, with a mean age of 33.41 ± 12.13 years at the first consultation, and the cohort showed a marked female predominance (74.13%), a distribution that is broadly consistent with the known epidemiology and healthcare-seeking patterns of anxiety and depressive disorders.

As shown in Table 1, anxiety and/or depressive diagnoses were recorded in 2140 patients, corresponding to 61.7% of the full cohort, while 43.8% of the full cohort presented anxiety–depression comorbidity.

### 3.2. Severity Distribution

To characterize the overall clinical burden captured by questionnaire-based assessment, symptom severity was first examined across all patients with available anxiety measures. As shown in Table 2, mild anxiety was the most frequent severity category (with 56.1%), accounting for more than half of the evaluable subgroup, whereas moderate and severe anxiety were progressively less common.

A comparable analysis was then conducted for depressive symptoms. As presented in Table 3, depression severity also showed a predominance of lower and intermediate severity levels, with mild and moderate categories together accounting for the great majority of patients with available questionnaire data. In contrast to anxiety, however, the distribution of depressive severity appeared somewhat more balanced between mild and moderate categories (with 43.5% and 40.4%, respectively).

**Figure 1:**
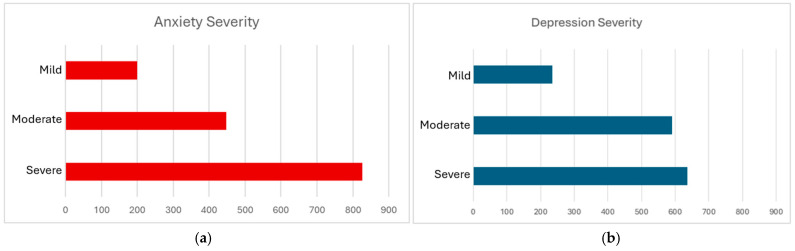
Distribution of symptom severity levels in the study cohort. (**a**) Distribution of anxiety severity categories among patients with available questionnaire data, classified as mild, moderate, or severe according to validated cut-off scores. (**b**) Distribution of depression severity categories based on validated cut-off scores. Values represent the number of patients within each severity category.

These overall distributions are visually summarized in Figure 1, which provides an intuitive representation of symptom-severity architecture across the study cohort. The graphical presentation highlights the predominance of mild-to-moderate symptom levels in both domains, while also showing that severe cases remain clinically relevant within the tele–mental health population. By presenting anxiety and depression side by side, the figure facilitates a direct comparison of severity structure between the two conditions and illustrates the broadly similar, although not identical, distributional profiles observed in the cohort.

### 3.3. Distribution of Symptom Severity According to Sex and Age

To further explore the demographic structure of anxiety severity in the cohort, symptom levels were stratified according to age group and sex. As shown in Table 4, anxiety symptoms were more frequently observed in females across almost all age categories, with the largest concentration of cases occurring between 20 and 49 years of age. Within this female subgroup, the 20–29 and 30–39-year intervals accounted for the greatest proportion of patients with anxiety symptoms, and substantial proportions of moderate to severe presentations.

In males, anxiety symptoms followed a broadly similar age-related pattern, although with lower absolute frequencies across all severity levels. The highest concentration of male cases was observed between 20 and 39 years of age, with a peak in the 30–39 group.

**Figure 2:**
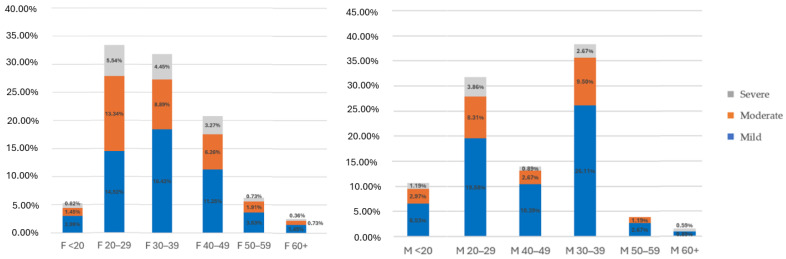
Distribution of anxiety severity according to age group and sex among patients with available questionnaire data. Graphical representation of the number of patients classified with mild, moderate, and severe anxiety symptoms across age groups and stratified by biological sex. Severity categories were derived from standardized questionnaire scores. Percentages are expressed relative to the total number of individuals within each sex category.

The age- and sex-related distribution of anxiety severity is illustrated graphically in Figure 2.

A parallel analysis was conducted for depressive symptoms to determine whether the demographic structure observed for anxiety was also present in depression. As summarized in Table 5, depressive symptoms showed a similarly marked predominance among females, again with the greatest concentration of cases observed in the 20–29 and 30–39-year age groups. These strata accounted for a substantial proportion of mild, moderate, and severe depressive presentations, indicating that depressive burden in this cohort was also particularly concentrated in early and middle adulthood.

In male patients, depression followed a comparable but less pronounced age distribution, with the highest frequencies observed between 20 and 39 years of age, especially in the 30–39 subgroup. Compared with anxiety, the depressive profile appears to show a somewhat stronger representation of moderate and severe cases within the most affected age intervals, suggesting that depressive symptoms may be more evenly distributed across intermediate and higher levels of severity.

**Figure 3:**
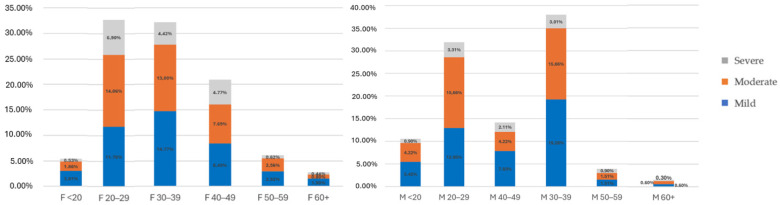
Distribution of depression severity according to age group and sex among patients with available questionnaire data. Graphical representation of the number of patients classified with mild, moderate, and severe depression symptoms across age groups and stratified by biological sex. Severity categories were derived from standardized questionnaire scores. Percentages are expressed relative to the total number of individuals within each sex category.

Figure 3 provides a visual summary of the age- and sex-specific distribution of depressive symptom severity across the cohort. By displaying severity categories across demographic strata, the figure makes clear that depression, like anxiety, is disproportionately represented among female patients and is concentrated in young and middle-adult age groups.

When stratified by age and sex, both anxiety and depression severity categories were more frequently represented among female patients, particularly between 20 and 49 years of age. Because the full cohort had a marked female predominance, these results should be interpreted primarily as descriptive distributions rather than evidence of sex-specific differences in severity risk. Normalized proportions within age–sex strata are provided to support comparison across demographic groups.

### 3.4. Symptom Severity and Anxiety–Depression Comorbidity

To examine whether anxiety severity differed according to diagnostic context, patients with isolated anxiety were compared with those presenting anxiety–depression comorbidity. As shown in Table 6, anxiety severity in the isolated-anxiety subgroup was predominantly mild, with only a very small number of patients classified in the moderate or severe categories. By contrast, patients with comorbid anxiety–depression accounted for the overwhelming majority of evaluable anxiety cases and showed a substantially broader distribution across severity levels, including a marked representation of moderate and severe symptomatology.

A comparable analysis was performed for depressive symptoms in order to determine whether the same diagnostic gradient was also observed for depression. As summarized in Table 7, isolated depression was represented by a very small subgroup and was largely concentrated in the mild range, whereas depressive symptoms in the context of anxiety–depression comorbidity showed a much broader severity profile. In particular, the comorbid group accounted for nearly all evaluable cases and included substantial proportions of moderate and severe depressive symptomatology, indicating that depression, like anxiety, tends to present with greater clinical intensity when embedded within a comorbid internalizing profile.

**Figure 4:**
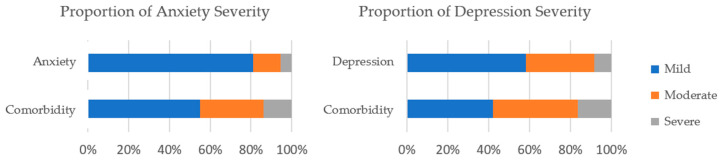
Severity distribution of anxiety and depression according to diagnostic status. Comparison of severity categories for anxiety and depressive symptoms between patients with isolated disorders and those presenting anxiety–depression comorbidity. Proportions represent the relative frequency of mild, moderate, and severe severity levels within each diagnostic group.

The comparative severity structure of isolated and comorbid presentations is summarized visually in Figure 4. By displaying anxiety and depression side by side according to diagnostic status, the figure makes immediately apparent that isolated disorders are predominantly characterized by mild symptom levels, whereas comorbid anxiety–depression is associated with a more substantial contribution from moderate and severe categories in both domains.

Patients with isolated anxiety or isolated depression were predominantly represented in the mild severity category, whereas patients with anxiety–depression comorbidity included a broader severity spectrum. Therefore, comparisons between isolated and comorbid diagnostic groups should be interpreted cautiously, as observed utilization differences may partly reflect differences in symptom severity distribution rather than comorbidity status alone.

### 3.5. Coupling Between Anxiety and Depression Severity in the Comorbid Subgroup

To further characterize the internal clinical structure of anxiety–depression comorbidity, the relationship between anxiety and depression severity levels was examined within the comorbid subgroup. As shown in Table 8, the cross-tabulation of severity categories revealed a highly organized distribution, with the greatest concentrations observed along the diagonal corresponding to concordant severity levels in both domains. In particular, mild anxiety was most frequently paired with mild depression, moderate anxiety with moderate depression, and severe anxiety with severe depression, indicating that symptom burden in one domain tended to be accompanied by a comparable degree of burden in the other. Rather, they appear to form a clinically coherent severity architecture in which both symptom domains tend to intensify together.

The chi-square analysis showed a statistically significant association between anxiety and depression severity categories within the comorbid subgroup (*p* = 5524 × 10^−107^). Concordant severity combinations occurred more frequently than expected under independence. This finding indicates that anxiety and depression severity were not randomly distributed within comorbid cases; however, because of the cross-sectional and retrospective nature of the analysis, it should not be interpreted as evidence of causal or mechanistic coupling.

To determine which severity combinations contributed most strongly to the overall association, adjusted standardized residuals were examined for each cell of the contingency table. As presented in Table 9, the most prominent positive residuals were observed for the combinations mild anxiety with mild depression, moderate anxiety with moderate depression, and severe anxiety with severe depression, indicating that these concordant pairings occurred more frequently than expected under independence. In contrast, several discordant combinations showed negative residuals, suggesting that mismatched severity levels were less common than would be expected by chance alone. Particularly noteworthy is the strong positive residual for the severe–severe combination, which supports the view that the highest-burden patients tend to cluster within a clearly identifiable subgroup of comorbid presentations.

### 3.6. Comorbidity Healthcare-Utilization Profiles Across Diagnostic and Severity Strata

#### 3.6.1. Consultation Frequency and Inter-Visit Interval

To examine whether healthcare utilization within the anxiety–depression comorbid subgroup varied according to sex, the mean number of consultations and the mean interval between consecutive visits were analysed separately for female and male patients. As shown in Table 10, female patients demonstrated a higher mean number of consultations than male patients, together with shorter inter-visit intervals. Overall, the mean consultation rate across the cohort was 30.18 days per consultation. Female patients showed a slightly lower consultation rate than males (29.57 vs. 32.25 days per consultation), indicating modestly more frequent follow-up after accounting for observation time. This combination suggests a pattern of greater care engagement and more closely spaced follow-up among women within the comorbid subgroup.

Age-stratified analyses were then performed to determine whether the operational profile of anxiety–depression comorbidity also varied across the lifespan. As presented in Table 11, consultation burden was concentrated primarily in adulthood, particularly between 20 and 49 years of age, where the mean number of consultations remained consistently high. The highest mean number of consultations, however, was observed in patients aged 60 years and older, suggesting that older adults with comorbid anxiety–depression may also represent a subgroup requiring sustained longitudinal follow-up. Inter-visit intervals showed an equally informative pattern. Patients younger than 20 years had relatively short intervals between consultations, consistent with a model of closer monitoring, while adults aged 30–49 years also showed comparatively tight follow-up spacing. Consultation rates were broadly similar across age groups, ranging from 29.05 days per consultation among patients aged 20–29 years to 32.27 days per consultation among those aged 60 years or older.

To determine whether the healthcare-utilization profile associated with anxiety–depression comorbidity differed from that observed in isolated disorders, consultation frequency, inter-visit intervals and consultation rate were compared across the three main diagnostic groups. As shown in Table 12, patients presenting anxiety–depression comorbidity exhibited the highest mean number of consultations, clearly exceeding the corresponding values observed in patients with anxiety alone and depression alone. In parallel, the comorbid group also showed a shorter mean inter-visit interval than the isolated-disorder groups, but it was the depression-alone group that had the lowest consultation rate with 28.91.

To explore whether healthcare utilization varied according to symptom burden within the anxiety–depression comorbid subgroup, consultation frequency and inter-visit intervals were stratified by anxiety severity. As shown in Table 13, meaningful differences emerged across severity categories, indicating that the operational profile of care was not homogeneous within comorbid presentations. Patients with mild anxiety had the lowest mean number of consultations, whereas those with moderate anxiety showed the highest consultation frequency. Patients with severe anxiety also maintained a high consultation burden and, notably, exhibited the shortest mean interval between visits, suggesting a pattern of more closely spaced follow-up as symptom intensity increased. Consultation rates were also compared according to anxiety severity. Mean values were 29.63, 30.24 and 30.77 days per consultation for mild, moderate and severe anxiety, respectively.

A complementary analysis was then conducted to determine whether healthcare-utilization patterns within the anxiety–depression comorbid subgroup also varied according to depression severity. As shown in Table 14, depressive symptom burden was likewise associated with differences in operational care metrics, indicating that utilization patterns within comorbid presentations were not solely shaped by anxiety severity, but also by the intensity of depressive symptoms. Patients with mild depressive symptoms showed the lowest consultation burden overall, whereas those with moderate and severe depression tended to require a greater number of consultations, supporting the interpretation that increasing depressive burden may be associated with more sustained engagement with care. Inter-visit intervals provided an additional layer of interpretive value. Patients with more severe depressive symptoms tended to show shorter spacing between visits than those with milder presentations, suggesting that higher depressive burden may be linked not only to a greater cumulative number of consultations, but also to a more closely structured follow-up schedule. In contrast, consultation rate showed a higher burden for moderate depression. Mean consultation rates were 28.10, 32.55 and 28.37 days per consultation for mild, moderate and severe depression, respectively.

For anxiety, severity-related differences in healthcare utilization were statistically significant (*p* = 0.0075), indicating that increasing symptom burden was associated with meaningful variation in consultation patterns. The most pronounced contrast was observed between the mild and moderate categories (*p* = 0.0038), suggesting that even transitions within the lower-to-intermediate range of severity may be reflected in measurable changes in care engagement. A comparable pattern was observed for depression, for which severity groups also differed significantly overall (*p* = 0.00063). Post hoc comparisons showed that the strongest contrasts were found between the mild and moderate categories (*p* = 0.0068) and between the mild and severe categories (*p* = 0.00078), further supporting the view that depressive burden is closely linked to differences in service utilization. By contrast, inter-visit intervals did not differ significantly across severity groups for either anxiety or depression (*p* = 0.8442 and *p* = 0.1479, respectively). However, analysing consultation rate, there weren’t any statistically significant differences between anxiety severity groups (*p* = 0.815). Post hoc comparisons confirmed the absence of significant pairwise differences between mild and moderate anxiety (*p* = 0.672), moderate and severe anxiety (*p* = 0.820), and mild and severe anxiety (*p* = 0.544). These findings suggest that previously observed differences in raw consultation counts according to anxiety severity are substantially attenuated when follow-up duration is taken into account. In contrast, analysing differences for consultation rates, a statistically significant difference between groups was revealed (*p* = 0.0043). Post hoc analyses identified significant differences between mild and moderate depression (*p* = 0.0029) and between moderate and severe depression (*p* = 0.0498), whereas mild and severe depression did not differ significantly (*p* = 0.858). Importantly, this pattern was non-linear, with moderate depression showing the highest mean number of days per consultation. Therefore, although depression severity remained associated with consultation rate after adjustment for follow-up duration, the relationship cannot be interpreted as a simple gradient of increasing healthcare utilization with increasing symptom severity.

#### 3.6.2. Appointment Duration

In addition to consultation frequency and inter-visit intervals, consultation duration was examined as a complementary operational metric within the anxiety–depression comorbid subgroup. As shown in Table 15, female patients had a longer mean consultation duration than male patients. The overall mean consultation duration in the comorbid subgroup was approximately 42 min. Differences in consultation duration were observed across demographic strata, although the magnitude of these differences was smaller than that observed for consultation counts and inter-visit intervals. Overall, consultation duration remained relatively stable across groups, indicating that variation in healthcare utilization within the comorbid subgroup was more evident in consultation frequency and follow-up spacing than in appointment length.

Consultation duration was also examined across age groups within the anxiety–depression comorbid subgroup. As shown in Table 16, mean appointment duration remained relatively stable across most age categories, with values clustering around the overall mean of approximately 42 min. No clear linear age-related trend was observed. Consultation duration was somewhat shorter among patients aged 40–49 years and somewhat longer among those aged 50–59 years; however, differences between age groups were modest overall. Compared with the variation observed in consultation frequency and inter-visit intervals, consultation duration showed a more homogeneous distribution across age strata. These findings indicate that appointment length remained broadly consistent across age groups within the comorbid subgroup.

To determine whether differences in healthcare utilization across diagnostic groups also extended to appointment length, mean consultation duration was compared between patients with anxiety–depression comorbidity, anxiety alone, and depression alone. As shown in Table 17, mean consultation duration was broadly similar across diagnostic categories, with only modest variation between groups. Patients with isolated anxiety had the longest mean consultation duration, followed by the comorbid subgroup, whereas patients with isolated depression had the shortest mean consultation time.

Overall, differences in consultation duration were smaller than those observed for consultation frequency and inter-visit intervals. Consultation duration remained relatively stable across diagnostic categories, indicating that variation in healthcare utilization was more evident in measures related to consultation frequency and follow-up spacing than in appointment length.

To further examine appointment-level healthcare utilization, consultation duration was analysed according to anxiety and depression severity. As shown in Table 18, mean consultation duration varied across severity categories for both symptom domains. For anxiety and depression, the longest mean consultation durations were observed in the moderate severity group, whereas mild and severe categories showed shorter average appointment times. For anxiety, overall differences in consultation duration between severity groups reached statistical significance, although pairwise post hoc comparisons were not statistically significant. For depression, a similar distribution of mean consultation duration was observed, with moderate severity showing the longest average appointments; however, overall differences between severity groups did not reach statistical significance. Compared with consultation frequency and inter-visit intervals, consultation duration showed a different pattern of variation across severity levels. Overall, differences in appointment length were relatively modest, indicating that consultation duration remained more stable across severity groups than other healthcare-utilization metrics examined in the study.

For anxiety, consultation duration differed significantly across severity groups overall (*p* = 0.0439), supporting the view that anxiety burden may influence the temporal structure of clinical encounters, although no individual pairwise contrasts remained significant in post hoc analyses. Therefore, these findings should be interpreted cautiously, as the overall effect was not supported by statistically significant pairwise group differences. For depression, the same general directional pattern was observed, but the overall group effect did not reach statistical significance (*p* = 0.4280).

### 3.7. Stability of Anxiety and Depression Severity Across Additional Psychiatric Comorbidity Status

**Figure 5:**
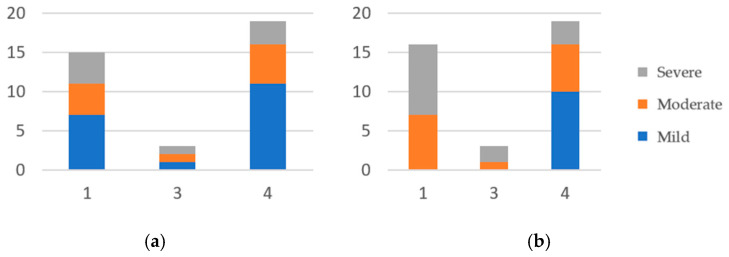
Distribution of anxiety and depression severity according to the presence of additional psychiatric comorbidities. (**a**) Anxiety severity distribution in patients with and without psychiatric comorbidities beyond anxiety–depression. (**b**) Depression severity distribution under the same conditions.

To further characterize the anxiety–depression comorbid subgroup, symptom severity distributions were examined according to the presence or absence of additional psychiatric comorbidities beyond anxiety and depression. As shown in Figure 5, the distribution of anxiety and depression severity categories was broadly similar across both groups. In both symptom domains, mild and moderate severity categories accounted for the largest proportion of cases, whereas severe presentations represented a smaller proportion of patients. No major shifts in the overall severity distribution were observed according to the presence of additional psychiatric comorbidities. Overall, the severity patterns identified within the anxiety–depression comorbid subgroup remained relatively consistent regardless of additional psychiatric comorbidity status.

## 4. Discussion

This study provides real-world evidence that anxiety–depression comorbidity in tele–mental health is both highly prevalent and clinically meaningful, being associated not only with a distinct severity architecture but also with a more intensive pattern of healthcare utilization. In this cohort, the study population was predominantly composed of young adults and showed a marked female predominance, while anxiety and/or depressive symptoms were highly prevalent overall. Nearly half of symptomatic patients presented anxiety–depression comorbidity, reinforcing the relevance of this dyad as a major clinical pattern in routine tele–mental health care. Although mild severity predominated in both domains, depressive symptoms showed a relatively greater representation of moderate-to-severe presentations than anxiety. Taken together, these findings are consistent with epidemiological literature showing that anxiety and depressive disorders frequently co-occur and contribute substantially to the global burden of mental illness [1,2,3,6]. The marked predominance of female patients, together with the concentration of cases in young adulthood, is also aligned with previous epidemiological evidence. Both anxiety and depressive symptoms were more frequently observed in women, with peak prevalence in the 20–29-year age group, whereas in men the highest frequencies were observed somewhat later, between 30 and 39 years. In addition, within corresponding age strata, women tended to present greater symptom severity than men. These findings are consistent with robust literature showing that women are disproportionately affected by both anxiety and depressive disorders across populations [12,13,14,15,16]. Multiple explanatory mechanisms have been proposed, including hormonal influences, sex differences in stress responsivity, psychosocial burden, and differing patterns of emotional processing and help-seeking behaviour [16,17]. In this context, our findings suggest that young adult women may represent a particularly vulnerable and high-burden subgroup within tele–mental health services. At the same time, the lower representation of the youngest and oldest age groups should be interpreted cautiously, as service access, digital engagement, and help-seeking behaviour may differ substantially at the extremes of age.

One of the most clinically relevant findings of the study was the severity structure associated with diagnostic status. Patients with anxiety–depression comorbidity showed a substantially greater proportion of moderate-to-severe symptomatology than those presenting isolated anxiety or isolated depression. This supports the interpretation that comorbidity reflects greater clinical burden rather than simple diagnostic coexistence. Such a pattern is consistent with previous work showing that comorbid anxiety and depressive disorders are associated with greater symptom load, poorer outcomes, higher relapse risk, and more pronounced functional impairment than single disorders [7,8,9,10,11]. In this sense, the present findings reinforce the value of examining anxiety and depression jointly in routine-care datasets which is consistent with dimensional models of internalizing psychopathology and suggests that anxiety and depression severity are closely associated within comorbid presentations.

Within the comorbid subgroup, anxiety and depression severity levels also showed a significant tendency to cluster in concordant categories. Mild anxiety was most often accompanied by mild depressive symptoms, while severe anxiety was strongly associated with severe depression. Although the magnitude of association was moderate, this pattern strongly suggests that symptom escalation in one domain tends to be accompanied by escalation in the other. This observation is highly compatible with dimensional models of internalizing psychopathology, in which anxiety and depression are conceptualized as closely related and partially overlapping manifestations within a broader affective spectrum [11]. Rather than functioning as fully independent symptom domains, they appear in this cohort to form a clinically coherent severity architecture.

Interestingly, the presence of additional psychiatric comorbidities beyond anxiety and depression did not substantially alter the observed severity distributions. Anxiety and depression severity patterns appeared broadly similar regardless of additional psychiatric diagnoses, suggesting that the association between the two symptom domains remained relatively stable across different levels of psychiatric complexity. Although the present analysis cannot determine the independent contribution of specific comorbid disorders, these findings indicate that the severity patterns observed within the anxiety–depression subgroup were not markedly modified by the presence of broader psychiatric multimorbidity.

The healthcare-utilization analyses add an important operational dimension to these clinical observations. Compared with isolated anxiety or depression, anxiety–depression comorbidity was associated with a distinctly higher-intensity pattern of care, characterized by more consultations and shorter inter-visit intervals. This suggests that the greater symptom burden associated with comorbidity translates into a more demanding follow-up structure in routine tele–mental health practice. Such a pattern is clinically plausible and consistent with previous studies showing that individuals with anxiety and mood disorders utilize mental health services more frequently than the general population [21]. From a service-delivery perspective, this result is particularly important because it links comorbidity directly to measurable operational demand, thereby moving beyond symptom description alone. Sex- and age-related patterns in healthcare utilization further refined this operational picture. Female patients required more consultations and had shorter intervals between visits than males, suggesting greater longitudinal service engagement and a potentially higher burden of ongoing care. This pattern may reflect not only greater symptom burden, but also known sex differences in help-seeking behaviour and continuity of mental health service use [15,16,17]. Age-stratified analyses showed that healthcare utilization remained substantial across most adult age groups, with certain subgroup-specific patterns of interest. Younger patients showed relatively fewer consultations but maintained reasonably close follow-up spacing, consistent with early engagement and active monitoring, while older adults exhibited the highest mean number of consultations and the shortest inter-visit intervals, suggesting closer and potentially more intensive follow-up in later life. Together, these findings support the view that healthcare-utilization profiles in tele–mental health are demographically structured rather than uniformly distributed. To address the potential influence of follow-up duration on healthcare-utilization measures, additional analyses used a follow-up-adjusted consultation-rate metric expressed as mean days per consultation. This approach allowed for a more standardized comparison of healthcare utilization across patients with different observation periods. After accounting for follow-up duration, some utilization differences became less pronounced, indicating that part of the variation observed in crude utilization metrics may have reflected differences in observation time rather than clinical characteristics alone. These findings highlight the importance of considering temporal factors when interpreting healthcare-utilization patterns in observational tele–mental health data and support the use of follow-up-adjusted metrics to better characterize patterns of healthcare engagement. Stratification by symptom severity also revealed clinically informative gradients in service use. For anxiety, moderate severity was associated with the highest number of consultations, whereas severe anxiety was associated with shorter inter-visit intervals. For depression, the pattern appeared somewhat more linear, with greater consultation frequency as severity increased and the shortest follow-up intervals in severe cases. However, follow-up-adjusted consultation-rate analyses produced a more nuanced picture. For anxiety, consultation rates did not differ significantly between severity groups, suggesting that some of the differences observed in crude utilization measures may have been influenced by variation in follow-up duration. For depression, consultation rate remained significantly associated with symptom severity, although the relationship was non-linear, with moderate depression showing the longest interval between consultations. Overall, these findings suggest that severity-related differences in healthcare utilization are more complex than a simple increase in service use with increasing symptom burden and that different utilization metrics may capture distinct aspects of clinical care.

An additional limitation concerns the possibility of confounding by symptom-severity distribution when comparing healthcare utilization between isolated anxiety/depression and anxiety–depression comorbidity. Although a sensitivity analysis within matched severity strata would have been informative, this was not feasible in the present dataset because the isolated diagnostic groups with available questionnaire-based severity data were very small, particularly in the moderate and severe categories. As a result, stratified comparisons between isolated and comorbid cases within the same severity level would have been underpowered and potentially unstable. Therefore, the observed differences in healthcare utilization should not be interpreted as reflecting comorbidity status alone, but rather as patterns that may partly reflect the higher symptom-severity burden observed in the comorbid group. Future studies with larger isolated-disorder subgroups are needed to disentangle the independent effects of diagnostic comorbidity and symptom severity on healthcare utilization.

Importantly, anxiety–depression comorbidity was not associated with longer consultations when compared with isolated disorders, suggesting that the distinctive operational burden of comorbidity is more strongly reflected in consultation frequency and continuity than in the length of each individual encounter. In addition, moderate symptom severity—particularly for anxiety—was associated with longer consultations than mild or severe categories. One plausible explanation is that moderate presentations may require more extensive diagnostic clarification, therapeutic adjustment, or exploratory clinical discussion, whereas severe cases may be managed through more frequent but not necessarily longer contacts. These findings highlight the multidimensional nature of healthcare utilization and suggest that different operational metrics may capture different aspects of clinical complexity.

Taken together, these results contribute to the growing literature examining the relationship between symptom burden and healthcare utilization in digital-care environments. Tele–mental health platforms generate structured clinical and operational datasets that make it possible to explore how symptom profiles are associated with patterns of service use over time. In the present study, symptom severity, comorbidity status, and healthcare-utilization measures showed consistent associations across several analyses. However, these findings should be considered descriptive and hypothesis-generating rather than definitive evidence of digital phenotypes.

This interpretation is particularly relevant because telemedicine has already demonstrated clinical effectiveness in the management of mood and anxiety disorders. Systematic reviews and meta-analyses indicate that tele–mental health interventions can produce clinically meaningful improvements in depressive and anxiety symptoms while improving access, continuity, and flexibility of care [18,20,22]. Although the present findings should be interpreted as descriptive and hypothesis-generating, they are consistent with broader person-centered approaches that seek to use routinely collected clinical data to characterize clinically meaningful patient profiles and better understand variation in healthcare needs [28]. Within this context, operational phenotypes derived from telehealth platforms may provide an additional layer of actionable information that complements symptom scales and supports more efficient care planning. Rather than replacing clinical judgment, such metrics may help identify patients who are likely to require more intensive follow-up, closer monitoring, or more sustained contact over time.

Some limitations should be considered when interpreting these findings. First, questionnaire completion was not universal among diagnosed patients a formal comparison between patients with and without available questionnaire data was not performed, which may have introduced response bias and limited the representativeness of the severity analyses. Second, the study relied on routinely collected real-world data, and residual confounding cannot be excluded. Third, different validated instruments were used to assess anxiety and depression severity, which may have introduced some heterogeneity despite the harmonization strategy adopted. Fourth, the high variability observed in inter-visit intervals and some consultation-duration measures suggests the presence of substantial dispersion and possible outliers that could not be fully standardized within the dataset. Fifth, the retrospective design precludes causal inference, and the observed associations should therefore be interpreted as descriptive and hypothesis-generating rather than causal. Finally, the generalizability of the findings to other tele–mental health systems or conventional in-person settings remains to be established. The relatively small number of severe anxiety and depression cases may have limited the statistical power of severity-stratified analyses. In addition, the most severe clinical presentations may be underrepresented in this cohort, as such patients are more likely to receive follow-up in specialized public mental-health services or to access emergency psychiatric care during periods of clinical deterioration. Moreover, the present analyses did not distinguish between psychiatry and psychology consultations. Because consultation duration, follow-up frequency, and clinical management strategies may differ between provider types, part of the observed variation in healthcare-utilization measures may reflect differences in service composition rather than patient characteristics alone. Furthermore, healthcare-utilization variables such as consultation counts, inter-visit intervals, and appointment duration may exhibit non-normal or overdispersed distributions. Although follow-up-adjusted analyses were incorporated to address differences in observation time, future studies may benefit from generalized count-data models, longitudinal approaches, and other robust statistical techniques specifically designed for healthcare-utilization outcomes. Despite these limitations, the study has several important strengths. It is based on a large real-world tele–mental health cohort, combines symptom-severity assessment with operational service-level metrics, and examines comorbidity, demographic factors, and healthcare utilization within a unified analytic framework. This integrated perspective makes it possible to move beyond simple prevalence estimates and toward a more clinically meaningful characterization of patient complexity and care demand.

Overall, our findings support the development of operational digital phenotypes of anxiety–depression comorbidity grounded in routine-care data. Symptom severity and comorbidity were not merely descriptive clinical labels; they were associated with consistent and measurable differences in healthcare utilization, particularly in consultation frequency and follow-up cadence. By integrating severity, comorbidity structure, age, sex, and service-level metrics, the study identifies reproducible resource-use signatures that may support data-informed triage, adaptive follow-up planning, and more efficient allocation of clinical resources. Importantly, although derived from tele–mental health records, this framework is not inherently platform-dependent. The linkage between symptom architecture and longitudinal utilization metrics may also be relevant to in-person care settings, where visit frequency, encounter timing, and monitoring intensity are equally measurable. In this sense, the present study demonstrates the feasibility of constructing clinically interpretable operational phenotypes in mental health care using routinely collected data, while also laying the groundwork for future predictive and longitudinal models aimed at prospective risk stratification.

## 5. Conclusions

This real-world study shows that anxiety–depression comorbidity in tele–mental health is not merely a diagnostic overlap, but a clinically meaningful presentation associated with measurable differences in healthcare utilization. Comorbid cases were highly prevalent in this cohort and displayed marked severity coupling, with greater symptom burden in one domain tending to co-occur with greater severity in the other. Importantly, these clinical patterns were reflected in operational care metrics, particularly consultation frequency and follow-up cadence. Beyond confirming the relevance of anxiety–depression comorbidity, our findings suggest that routinely collected telehealth data can support the identification of clinically interpretable resource-use signatures. Patients with comorbid and more severe symptom profiles tended to require more intensive follow-up, with especially pronounced utilization patterns in young adult women, while older adults showed evidence of closer monitoring despite differing overall consultation profiles. Taken together, these results support the view that symptom architecture, demographic characteristics, and healthcare-utilization variables can be meaningfully integrated within a pragmatic digital-phenotyping framework.

Overall, the present study demonstrates that anxiety–depression comorbidity is highly prevalent within routine tele–mental health practice and is associated with distinct patterns of symptom severity and healthcare utilization. The observed associations between comorbidity, severity, and operational care metrics suggest that routinely collected telehealth data can provide valuable insights into real-world patterns of service use. However, follow-up-adjusted analyses showed that some utilization differences became attenuated after accounting for observation time, particularly for anxiety severity. Consequently, these findings should be interpreted as descriptive and hypothesis-generating rather than as definitive evidence of digital phenotypes. Future longitudinal studies incorporating treatment variables, repeated symptom assessments, and clinical outcomes will be necessary to determine the stability, predictive value, and practical relevance of these observed utilization patterns.

## Figures and Tables

**Table 1:** Distribution of clinician-recorded diagnostic categories in the full cohort: anxiety only, depression only, anxiety–depression comorbidity, and absence of both of these conditions (none). Values represent the absolute number of patients and corresponding percentages within the total cohort.

Pathology	Number of Patients (%)
Anxiety and/or depression	2140 (61.7%)
-only anxiety	399 (11.5%)
-only depression	221 (6.4%)
-anxiety–depression comorbidity	1520 (43.8%)
None	1327 (38.3%)
Total	3467 (100%)

**Table 2:** Distribution of anxiety severity levels among patients with available questionnaire data. Anxiety symptom severity categorized into mild, moderate, and severe levels based on validated questionnaire cut-offs. Values represent the number and proportion of patients in each severity category.

Anxiety Severity	Number of Patients (%)
Mild	827 (56.1%)
Moderate	447 (30.3%)
Severe	199 (13.5%)
Total	1473 (100%)

**Table 3:** Distribution of depression severity levels among patients with available questionnaire data. Depressive symptom severity categorized into mild, moderate, and severe levels based on validated questionnaire cut-offs. Values represent the number and proportion of patients in each severity category.

Depression Severity	Number of Patients (%)
Mild	637 (43.5%)
Moderate	591 (40.4%)
Severe	235 (16.1%)
Total	1463 (100%)

**Table 4:** Distribution of anxiety severity according to age group and sex among patients with available questionnaire data. Cross-tabulation of anxiety symptom severity categories across age groups and stratified by biological sex. Percentages represent the proportion of patients relative to the total sample with anxiety severity data.

	Anxiety Severity				
Sex	Age	Mild (%)	Moderate (%)	Severe (%)	Total (%)
Female	<20	36 (2.44%)	16 (1.09%)	9 (0.61%)	61 (4.14%)
	20–29	162 (11.00%)	149 (10.12%)	62 (4.21%)	373 (25.32%)
	30–39	215 (14.60%)	99 (6.72%)	49 (3.33%)	363 (24.64%)
	40–49	130 (8.83%)	70 (4.75%)	36 (2.44%)	236 (16.02%)
	50–59	42 (2.85%)	22 (1.49%)	8 (0.54%)	72 (4.89%)
	60+	19 (1.29%)	8 (0.54%)	4 (0.27%)	31 (2.10%)
Male	<20	22 (1.49%)	10 (0.68%)	4 (0.27%)	36 (2.44%)
	20–29	66 (4.48%)	28 (1.90%)	13 (0.88%)	107 (7.26)
	30–39	88 (5.97%)	32 (2.17%)	9 (0.61%)	129 (8.76%)
	40–49	35 (2.38%)	9 (0.61%)	3 (0.20%)	47 (3.19%)
	50–59	9 (0.61%)	4 (0.27%)	-	13 (0.88%)
	60+	3 (0.20%)	-	2 (0.14%)	5 (0.34%)
Total		827 (56.14%)	447 (30.35%)	199 (9.78%)	1473 (100%)

**Table 5:** Distribution of depression severity according to age group and sex among patients with available questionnaire data. Cross-tabulation of depressive symptom severity categories across age groups and stratified by biological sex. Percentages represent the proportion of patients relative to the total sample with depression severity data.

	Depression Severity				
Sex	Age	Mild (%)	Moderate (%)	Severe (%)	Total (%)
Female	<20	34 (2.32%)	21 (1.44%)	6 (0.41%)	61 (4.17%)
	20–29	133 (9.09%)	159 (10.87%)	78 (5.33%)	370 (25.29%)
	30–39	167 (11.41%)	147 (10.05%)	50 (3.42%)	364 (24.88%)
	40–49	95 (6.49%)	87 (5.95%)	54 (3.69%)	236 (16.13%)
	50–59	33 (2.26%)	29 (1.98%)	7 (0.48%)	69 (4.72%)
	60+	17 (1.16%)	9 (0.62%)	5 (0.34%)	31 (2.12%)
Male	<20	18 (1.23%)	14 (0.96%)	3 (0.21%)	35 (2.39%)
	20–29	43 (2.94%)	52 (3.56%)	11 (0.75%)	106 (7.45%)
	30–39	64 (4.37%)	52 (3.56%)	10 (0.68%)	126 (8.61%)
	40–49	26 (1.78%)	14 (0.96%)	7 (0.48%)	47 (3.21%)
	50–59	5 (0.34%)	5 (0.34%)	3 (0.21%)	13 (0.89%)
	60+	2 (0.14%)	2 (0.14%)	1 (0.07%)	5 (0.34%)
Total		637 (43.54%)	591 (40.40%)	235 (16.06%)	1463 (100%)

**Table 6:** Distribution of anxiety severity levels according to diagnostic status among patients with available questionnaire data. Comparison of anxiety symptom severity between patients with isolated anxiety and those presenting anxiety–depression comorbidity. Values represent the number and percentage of patients with available questionnaire data and anxiety diagnostics.

	Anxiety Severity			
Pathology	Mild (%)	Moderate (%)	Severe (%)	Total (%)
Only Anxiety	30 (2.05%)	5 (0.34%)	2 (0.14%)	37 (2.53%)
Comorbidity	784 (53.70%)	442 (30.27%)	197 (13.49%)	1423 (97.47%)
Total	814 (55.75%)	447 (30.62%)	199 (13.63%)	1460 (100%)

**Table 7:** Distribution of depression severity levels according to diagnostic status among patients with available questionnaire data. Comparison of depressive symptom severity between patients with isolated depression and those presenting anxiety–depression comorbidity. Values represent the number and percentage of patients with available questionnaire data and depression diagnostics.

	Depression Severity			
Pathology	Mild (%)	Moderate (%)	Severe (%)	Total (%)
Only Depression	7 (0.49%)	4 (0.27%)	1 (0.07%)	12 (0.84%)
Comorbidity	599 (41.83%)	587 (40.99%)	234 (16.34%)	1420 (99.16%)
Total	606 (42.32%)	591 (41.27%)	235 (16.41%)	1432 (100%)

**Table 8:** Contingency table showing the relationship between anxiety and depression severity levels in patients with anxiety–depression comorbidity. Cross-tabulation of anxiety severity (rows) and depression severity (columns) within the comorbid subgroup. Values represent the number and percentage of patients with available questionnaire data and anxiety–depression comorbidity.

	Depression Severity			
Anxiety Severity	Mild (%)	Moderate (%)	Severe (%)	Total (%)
Mild	492 (34.67%)	258 (18.18%)	32 (2.26%)	782 (55.11%)
Moderate	89 (6.27%)	256 (18.04%)	95 (6.69%)	440 (31.01%)
Severe	18 (1.27%)	72 (5.07%)	107 (7.54%)	197 (13.88%)
Total	599 (42.21%)	586 (41.30%)	234 (16.49%)	1419 (100%)

**Table 9:** Adjusted standardized residuals for the anxiety–depression severity contingency analysis. Adjusted standardized residuals from the chi-square test examining the relationship between anxiety and depression severity levels in patients with anxiety–depression comorbidity with available questionnaire data. Cells highlighted in green indicate values greater than +2 (more frequent than expected), while cells highlighted in red indicate values lower than −2 (less frequent than expected).

	Depression Severity		
Anxiety Severity	Mild (%)	Moderate (%)	Severe (%)
Mild	8.91	−3.61	−8.54
Moderate	−7.10	5.51	2.63
Severe	−7.15	−1.04	13.07

**Table 10:** Mean consultation frequency, inter-visit intervals and consultation rate according to sex among patients with anxiety–depression comorbidity. Mean number of consultations per patient and mean interval between consecutive consultations (in days) stratified by biological sex within the comorbid subgroup. Values are presented as mean ± standard deviation.

Sex	Mean Number of Visits	Mean Inter-Visit Interval (Days)	Mean Consultation Rate (Days/Visit)
Female	9.96 ± 9.69	51.54 ± 218.11	29.57 ± 25.04
Male	8.31 ± 8.07	70.86 ± 493.24	32.25 ± 25.02
Total	9.59 ± 9.37	55.89 ± 302.59	30.18 ± 25.37

**Table 15:** Consultation duration according to sex in the comorbid subgroup. Mean duration of tele-mental health consultations (minutes) stratified by biological sex among patients presenting anxiety–depression comorbidity. Values are presented as mean ± standard deviation.

Sex	Mean Appointment Duration (Minutes)
Female	43.24 ± 94.01
Male	37.73 ± 24.75
Total	42.00 ± 83.61

**Table 16:** Consultation duration across age groups in the comorbid subgroup. Mean appointment duration (minutes) stratified by age group among patients with anxiety–depression comorbidity. Values represent mean ± standard deviation.

Age	Mean Appointment Duration (Minutes)
<20	41.74 ± 52.18
20–29	41.43 ± 55.19
30–39	43.89 ± 128.49
40–49	38.89 ± 24.50
50–59	45.74 ± 65.03
60+	40.69 ± 26.59
Total	42.00 ± 83.61

**Table 17:** Mean consultation duration according to diagnostic category. Comparison of mean teleconsultation duration (minutes) among patients with anxiety–depression comorbidity, anxiety alone, and depression alone.

Pathology	Mean Appointment Duration (Minutes)
Comorbidity	41.998 ± 83.61
Only Anxiety	44.84 ± 103.95
Only Depression	39.29 ± 38.21
Total	42.25 ± 84.42

**Table 18:** Consultation duration stratified by symptom severity for anxiety and depression in the comorbid subgroup with available questionnaire data. Mean duration of tele-mental health consultations (minutes) according to symptom severity category for anxiety and depressive symptoms.

	Mean Appointment Duration (Minutes)	
Severity	Anxiety	Depression
Mild	39.02 ± 31.51	40.71 ± 42.33
Moderate	50.56 ± 148.46	45.55 ± 126.31
Severe	35.97 ± 14.24	37.68 ± 25.34
Total	42.19 ± 86.27	42.21 ± 86.36

**Table 11:** Mean consultation frequency, inter-visit intervals and mean consultation rate across age groups among patients with anxiety–depression comorbidity. Mean number of consultations and mean inter-visit interval (days) stratified by age group within the comorbid subgroup. Values represent mean ± standard deviation.

Age	Mean Number of Consultations	Mean Inter-Visit Interval (Days)	Mean Consultation Rate (Days/Visit)
<20	7.65 ± 6.07	41.27 ± 38.71	31.3 ± 25.94
20–29	9.49 ± 9.18	67.62 ± 449.14	29.05 ± 24.92
30–39	9.76 ± 9.56	41.97 ± 43.14	30.91 ± 24.35
40–49	9.77 ± 9.80	40.97 ± 49.41	30.15 ± 27.21
50–59	9.32 ± 9.82	76.69 ± 238.56	30.36 ± 28.37
60+	12.78 ± 11.12	34.75 ± 19.09	32.27 ± 21.71
Total	9.59 ± 9.37	55.89 ± 302.59	30.18 ± 25.37

**Table 12:** Comparison of healthcare utilization according to diagnostic status. Mean number of visits, mean inter-visit interval (days) and mean consultation rate (days/visit) across diagnostic groups including anxiety–depression comorbidity, anxiety alone, and depression alone. Values are expressed as mean ± standard deviation.

Pathology	Mean Number of Visits	Mean Inter-Visit Interval (Days)	Mean Consultation Rate (Days/Visit)
Comorbidity	9.59 ± 9.37	55.89 ± 302.56	30.17 ± 25.37
Only Anxiety	6.64 ± 6.83	174.48 ± 2313.55	30.35 ± 30.27
Only Depression	6.52 ± 6.00	74.23 ± 499.02	28.91 ± 21.80
Total	8.72 ± 8.75	79.72 ± 1039.91	28.79 ± 25.91

**Table 13:** Mean number of visits, mean inter-visit interval (days) and mean consultation rate (days/visit) stratified by anxiety severity in the comorbid subgroup with available questionnaire data.

Anxiety Severity	Mean Number of Visits	Mean Inter-Visit Interval (Days)	Mean Consultation Rate (Days/Visit)
Mild	8.91 ± 8.69	53.45 ± 9.22	29.63 ± 22.49
Moderate	10.54 ± 9.78	50.52 ± 203.83	30.24 ± 26.99
Severe	10.20 ± 9.76	40.87 ± 47.93	30.77 ± 27.31
Total	9.60 ± 9.22	50.79 ± 271.67	29.98 ± 24.64

**Table 14:** Mean number of visits, inter-visit interval (days) and mean consultation rate (days/visit) stratified by depression severity in the comorbid subgroup with available questionnaire data.

Depression Severity	Mean Number of Visits	Inter-Visit Interval (Days)	Mean Consultation Rate (Days/Visit)
Mild	8.58 ± 8.35	40.18 ± 71.87	28.10 ± 19.83
Moderate	9.99 ± 9.56	67.57 ± 416.02	32.55 ± 30.45
Severe	11.11 ± 10.15	36.18 ± 25.73	28.37 ± 18.25
Total	9.58 ± 9.22	50.84 ± 271.95	29.99 ± 24.66

## Data Availability

The original contributions presented in this study are included in the article. Further inquiries can be directed to the corresponding author.
